# Some American Hospitals

**Published:** 1892-06-04

**Authors:** 


### WITHIN THE HOSPITALS.

### SOME AMERICAN HOSPITALS.

In the second of the articles of Our Special Commissioner under this title which appeared in The Hospital of January 9th of the present year, the City and County Hospital, San Francisco, was very severely criticised. Our Special Commissioner said with force and truth:—

No stranger who is adequately informed could visit San Francisco at the present time without a feeling of sorrow and regret, that here, under present conditions the lot of the sick and needy is anything but satisfactory.

In the course of his report, Our Special Commissioner made an earnest appeal to the press of San Francisco to combine to arouse and fix public attention to the sufferings of the sick poor of the city and the neglectful conditions which at present characterise the arrangements for their accommodation. This appeal has been responded to with a heartiness which reflects great credit upon our confrères in San Francisco. The article we published has been reproduced in the papers, and members of the staff of the *San Francisco Chronicle*, have been sent to interview members of the Board of Health, and to ascertain the truth about the City and County Hospital. In the result the *Chronicle* describes it as:—"A Decrepit Disgrace." "The Dilapidated County Hospital," with "Rickety Wards, Foul Closets, Rain in the Soup, and No Eggs." They pay us the compliment of describing us as "A famous English Sanitary magazine," and say that both Dr. Regensberger, and Dr. Long, of the Board of Health, confirmed the statements very vigorously when interviewed, and declared that the City and County Hospital had been aptly compared to a wretched workhouse. Dr. Regensberger said:—

I regard the hospital as a disgrace to the city; as an outrage on the taxpayers of the city; as a senile apology for an infirmary for the unfortunate sick. Hampered by a miserably insufficient annual appropriation that is thickly covered with an insufferable quantity of red, red tape, the present Board of Health is trying its best, in the face of this bugbear, to remedy the long-standing wretchedness that haunts the palsied rafters and bleak rooms of the County Hospital. While engaged in this unequal work, a great sanitary journal penetrates the infamy and scores it. All I can say is that the strictures published are substantially true. The City and County Hospital is a back number, and all the varnish in the world would not brighten its dull walls, nor would tons of the finest perfumery remove the stenches that breed in every cranny of the structure. I believe that the present management is doing everything possible to keep it clean, but the value of the labour is nil, for the flooring and beams are worn and rotted from age.

Dr. Cole, who was one of the builders of the Hospital, told me recently that it had been originally decided that the Hospital should be so constructed as to last fifteen years. He thought at the end of that time it would be torn down and rebuilt on broader lines to meet the larger demands of the population. That was why the pavilion style of architecture was employed and that was why inferior building materials were used. Twenty years have passed; it stands, but that's all it does do, and on stormy nights the patients come as near being "rocked in the cradle of the deep" as they could expect to be on dry land.

The fact of the matter is, we want a new hospital. Necessity and common humanity demand it. A man with a heart as large as a marble would say the same thing if he visited the wards and looked into the faces of sufferers, huddled pell-mell together in convict beds. We want a new hospital, supplied with modern conveniences which will add virtue to the physicians' work; a hospital supplied with electric call bells, improved bath-rooms, decent plumbing, plenty of ventilation, and lots of sunlight. And we want a decent appropriation—not less than 125,000 dollars—to run it with. What's the use of maintaining a hospital if we haven't enough money to properly feed the inmates? A chicken is a rarity to the hospital patients; milk is looked upon with abject veneration, eggs are scarcer than honest politicians. All the jugglery of materia medica isn't worth a copper if proper medicinal foods cannot be obtained to help the patients along towards recovery. A hospital is designed to cure sick people, not to kill them, and it's a pity this simple fact is not better impressed on the minds of the governing body.

Along with the other members of the Board of Health I visited the hospital yesterday. What I saw astonished and horrified me. The space between the bottom flooring of the building and the ground is filled up with deposits of decomposed organic matter which proceed from various leakages. The plumbing throughout the building is woefully deficient. Pipes are bursting constantly. Several of the upper wards have such unsubstantial foundations that they have to be propped up with cross-beams. The closets and bath-rooms emit nauseous odours. One of the crying needs of the hospital is a separate ward for demented patients, duly padded and barred for their especial reception. As I said before, the Board of Health is badly hampered on account of the insufficient annual appropriation. We need all the money we can get, and we need it for the unfortunate men and women who are sick. Now there are four visiting surgeons and physicians to the hospital, who each receive 100 dols. a month for two hours' daily work. They are professors in medical schools, and the hospital practice thus given them is invaluable to their students. This work ought to be done without salary; the practice offered and the other advantages are sufficient inducement, and the 400 dols. thus saved could be applied to the benefit of the patients.

When I inspected the hospital, I observed that the roof leaked in several places, notably in the kitchen and laundry. The laundry-man has to choose sunny days for his ironing to dodge the downpour, and the cook tries his best to keep the rain out of the soup pots.

It is refreshing to find that defects so patent when pointed out by an independent and impartial stranger should be received in San Francisco in the spirit in which they have been made. Active steps have, we understand, been taken to secure the demolition of the present hospital buildings, and we can only hope that a suitable site will be found nearer the centre of the town, and that upon it will be erected a hospital replete with every modern feature, constructive and sanitary, so that San Francisco may be able to claim that it is not behind other cities, either in charity or Christian care for the sick and suffering poor of one of the most beautiful cities of the world. We shall be very glad if our contemporaries in San Francisco will kindly keep us informed from time to time of the steps taken to secure adequate and proper hospital accommodation for the poor of the capital of California.

